# The consequences of viral infection on host DNA damage response: a focus on SARS-CoVs

**DOI:** 10.1186/s43141-022-00388-3

**Published:** 2022-07-13

**Authors:** Asmaa S. Mekawy, Zina Alaswad, Aya A. Ibrahim, Ahmed A. Mohamed, Abdelrahman AlOkda, Menattallah Elserafy

**Affiliations:** 1 Center for Genomics, Helmy Institute for Medical Sciences, Zewail City of Science and Technology, 12578 Giza, Egypt; 2 University of Science and Technology, Zewail City of Science and Technology, Giza, 12578 Egypt; 3 Department of Neurology and Neurosurgery, McGill University, Montreal, Quebec Canada; 4 Metabolic Disorders and Complications Program and Brain Repair and Integrative Neuroscience Program, Research Institute of the McGill University Health Centre, Montreal, Quebec Canada

**Keywords:** SARS-CoV, SARS-CoV-2, DNA damage response (DDR), DNA repair, COVID-19, Genomic instability

## Abstract

DNA damage and genome instability in host cells are introduced by many viruses during their life cycles. Severe acute respiratory syndrome coronaviruses (SARS-CoVs) manipulation of DNA damage response (DDR) is an important area of research that is still understudied. Elucidation of the direct and indirect interactions between SARS-CoVs and DDR not only provides important insights into how the viruses exploit DDR pathways in host cells but also contributes to our understanding of their pathogenicity. Here, we present the known interactions of both SARS-CoV and SARS-CoV-2 with DDR pathways of the host cells, to further understand the consequences of infection on genome integrity. Since this area of research is in its early stages, we try to connect the unlinked dots to speculate and propose different consequences on DDR mechanisms. This review provides new research scopes that can be further investigated in vitro and in vivo, opening new avenues for the development of anti-SARS-CoV-2 drugs.

## Background

Coronaviruses belong to the *Coronaviridae* family, order *Nidovirales*. They are characterized by a single-stranded positive-sense RNA genome, which contains 26 to 32 kilobases (kb) [1]. The severe acute respiratory syndrome coronavirus 2 (SARS-CoV-2) genome consists of 12 functional open reading frames (ORFs) with a total of ~30,000 nucleotides. Its 5′-terminal ORF1a/b of the genome codes for polyproteins 1a/1ab (pp1a/pp1ab), which are cleaved by proteases into 16 nonstructural proteins (nsps) [2]. The last third of its genome codes for four main structural proteins: spike (S), envelope (E), nucleocapsid (N), and membrane (M) proteins [3]. SARS-CoV-2 shares high homology with SARS-CoV on both the genomic and proteomic levels; however, they differ in ORF1a/b, ORF7b, ORF8, and S genes' sequences. SARS-CoV-2 additionally bears two proteins, ORF8 and ORF10, which are not present in SARS-CoV [4]. Interestingly, the shared proteins of both viruses show similar localization patterns in HeLaM cells [5].

Viruses have a limited coding capacity due to their small genome size. Therefore, they utilize host cellular factors and machineries to facilitate their replication and generation of progeny. Accordingly, numerous cellular pathways including DNA damage response (DDR) are manipulated as a consequence of viral infection [6]. DDR comprises complex signaling pathways that protect and maintain genomic integrity from endogenous and exogenous DNA damaging agents [7]. During the course of infection, DDR machineries could be recruited to the viral replication centers, or their signaling cascade could also be suppressed through various approaches such as nucleocytoplasmic shuttling of host factors [8]. Viral proteins could also directly interact with DDR pathways, affecting the cells’ repair capabilities. Such viral-host protein interactions induce genomic instability, which are often associated with the viral pathogenesis [9]. A recent article has also proposed that during SARS-CoV-2 infection, importing the host RNA binding proteins (RBPs) into the nucleus is reduced, which possibly results in R-loops formation. At a late stage of infection, R-loops could accumulate in the cell and overwhelm the DNA repair machinery causing DNA damage [10]. Notably, DNA damage caused by telomere dysfunction or other extracellular damaging agents facilitates SARS-CoV-2 entry through the upregulation of ACE2 expression [11].

Viruses also generally influence the host cell cycle progression to safeguard their replication. This affects the host DNA replication and repair checkpoints and causes cell cycle perturbations. For example, coronavirus infectious bronchitis virus (IBV) induces S and G2/M cell cycle arrest [12], and SARS-CoV induces G0/G1 and S-phase arrest [12, 13]. Moreover, SARS-CoV-2 infection leads to S/G2 phase arrest to ensure the abundance of nucleotides and facilitate the translocation of essential cellular factors for viral replication from the host nucleus to the site of replication in the cytoplasm [14].

Although manipulation of DDR by RNA viruses plays a substantial role in their pathogenesis, the mechanisms are not widely studied in a way similar to DNA viruses [9]. This review focuses on the known and proposed interactions of SARS-CoV and SARS-CoV-2 with DDR pathways to gain insights into the molecular implications on the host cell and its genome integrity. We also link what has been reported for other viruses to SARS-CoVs to propose potential consequences that could be further validated in vitro and in vivo.

### The interaction between SARS-CoV and the host DDR

#### Polymerase δ interacts with SARS-CoV nsp13

Pol δ plays a central role in genomic replication, especially in the lagging strand and Okazaki fragments maturation. It also has a proofreading activity to increase the replication fidelity [15]. The evolutionarily conserved p125 subunit, encoded by *POLD1* gene, is responsible for the essential catalytic 5′–3′ DNA polymerase and 3′–5′ exonuclease activities of Pol δ. The polymerase also contains three other smaller subunits coded by the *POLD2*, *POLD3*, and *POLD4* genes. The subunits together with the replication factor C and proliferating nuclear cell antigen (PCNA) form a polymerase holoenzyme complex [16]. Pol δ functions in various repair pathways including nucleotide excision repair (NER), mismatch repair (MMR), and base excision repair (BER) [17–19].

**Fig. 1:**
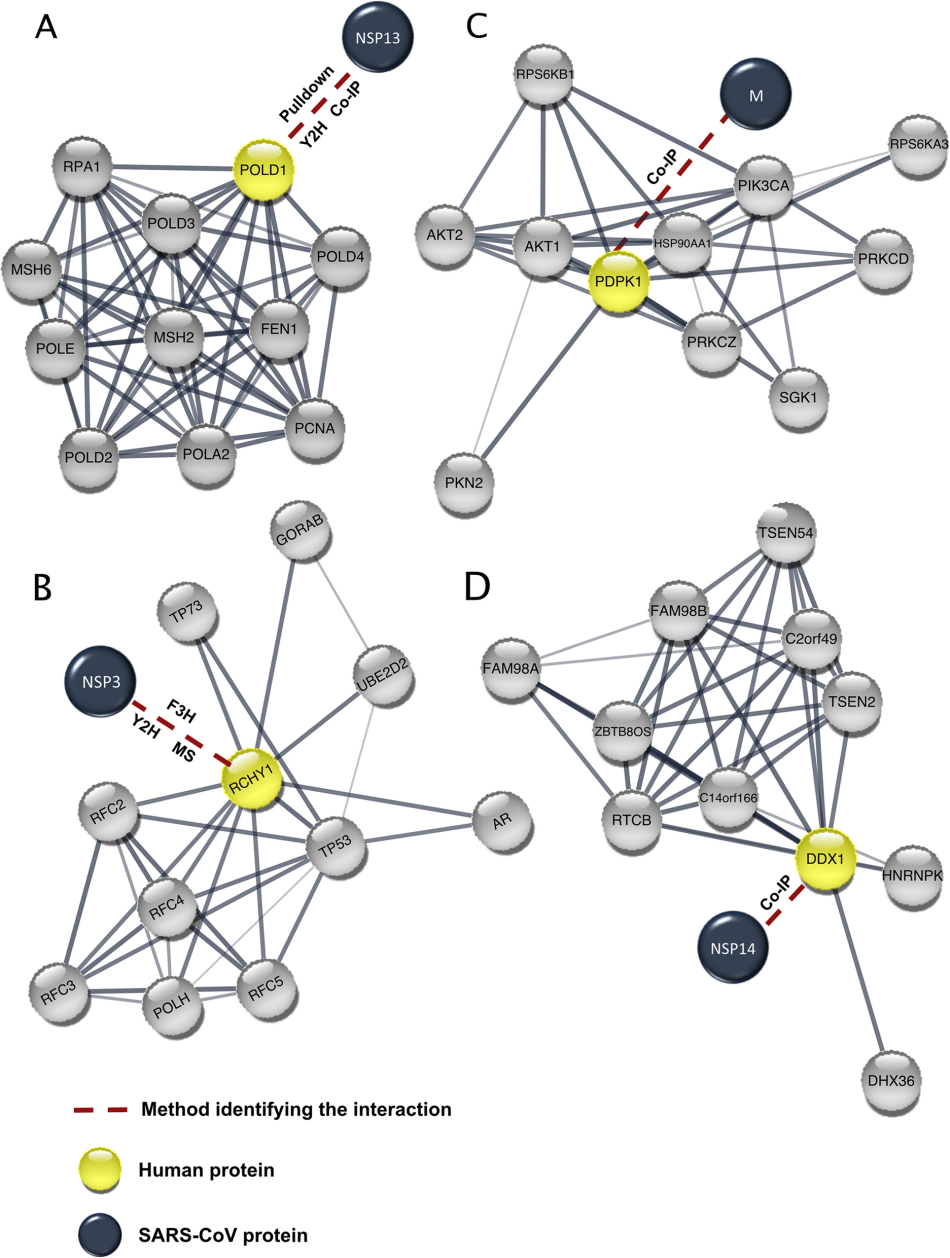
SARS-CoV protein interactions with the human DDR-associated proteins. **A**-**D**) The viral proteins are linked to the interaction partner represented in a protein-protein association network retrieved from STRING [26]. Y2H, yeast two-hybrid; Co-IP, co-immunoprecipitation; MS, mass spectrometry; F3H, fluorescence-3-hybrid assay

An interaction between the nonstructural protein 13 (nsp13) of SARS-CoV and Pol δ was reported, hinting at various possible consequences on the pathways that the polymerase is involved in. A yeast two-hybrid (Y2H) screen firstly showed an interaction between the C-terminus of p125 and nsp13, which was further confirmed via glutathione S-transferase (GST) pull-down and co-immunoprecipitation assays (Co-IP) (Table 1) [12]. nsp13 is a member of the helicase superfamily 1 that unwinds the double-stranded DNA or RNA in a 5′ to 3′ direction [20]. It is part of the viral replication and transcription complex (RTC), which plays a pivotal role in the life cycle of SARS-CoV [21]. Furthermore, the RNA 5′-triphosphatase activity of nsp13 proposes a vigorous role in the viral RNA 5′ capping [22]. nsp13 interaction with Pol δ results in a cell cycle arrest in the S-phase. Although the exact mechanism is yet to be understood, it is proposed that this interaction could result in a partial shift of Pol δ from the nucleus to the cytoplasm, which can consequently result in slow replication of the lagging strands, generation of single-stranded DNA (ssDNA) breaks, and eventually replication cessation. These events would result in the recruitment of ataxia telangiectasia and Rad3-related (ATR) to phosphorylate checkpoint kinase-1 (CHK1) and H2AX to stabilize the arrested forks [12]. The proposed mechanism is likely to exist in SARS-CoV-2, as nsp13 shows a 100% sequence similarity in both CoV and CoV-2 (Table 1) [23, 24]. In addition, recent reports could show an upregulation of ATR expression and enhanced phosphorylation of both CHK1 and H2AX in African green monkey kidney cells (Vero E6) infected with SARS-CoV-2 [25]. Further investigations are necessary to determine the localization of Pol δ, its behavior upon infection, and the consequences on interacting proteins represented in Fig. 1A.

**Table 1:** A summary of SARS-CoV proteins that interact with the host DDR

SARS-CoV protein	Function	Interacting host protein	Assays used to determine the interaction	Protein sequence similarity between SARS-CoV and SARS-CoV-2 according to [24]	Protein sequence similarity between SARS-CoV and SARS-CoV-2 according to [23]
Membrane protein (M protein)	Viral assembly	PDPK1	Co-IP	96.4%	98.2%
nsp3	Viral replication and transcription	RCHY1	Y2H F3H MS	86.5%	91.8%
nsp13	Helicase (Viral replication and transcription)	Polymerase δ	Y2H Pulldown Co-IP	100.0%	100.0%
nsp14	Guanine N7-methyltransferase and 3′–5′ exoribonuclease activity (viral replication fidelity)	DDX1	Co-IP	98.7%	99.1%

#### RCHY1 association with SARS-CoV nsp3

nsp3 is a 213 kDa glycosylated transmembrane multidomain protein acting together with multiple nsps, especially nsp4 and nsp6, to drive the replication and transcription processes through a suggested scaffolding function [27]. The SARS-unique domain (SUD) and the papain-like protease (PL^pro^) domain of nsp3 were found to interact with and stabilize the “Ring finger and CHY zinc finger domain-containing 1 (RCHY1)” human protein. The interaction was detected in a Y2H screen and confirmed by mass spectrometry (MS) and the fluorescence-3-hybrid (F3H) assay [28]. RCHY1 has an E3-dependent ubiquitination activity and contributes to proteasomal degradation of several proteins including the tumor suppressor p53, to regulate homeostasis of the cells. Additionally, RCHY1 monoubiquitinates the translesion DNA polymerase POLH, inhibiting its DNA damage bypass activity in the S-phase. Since RCHY1 impacts different pathways, interacts with key proteins (Fig. 1B), and regulates cell cycle progression, significant effects are expected upon its interaction with viral proteins [29]. The known consequences so far include an increase in the RCHY1-mediated p53 degradation [28]. This consequently can affect cell cycle progression and influences the activity of numerous DNA-repair pathways [30]. The target degradation of p53 also enhances SARS-CoV replication as p53 acts as a host antiviral factor that enhances the immune response and downregulates the viral replication [28, 31]. Interestingly, some viruses manipulate p53 levels in the cell either through upregulating or downregulating the expression level according to the virus’s life cycle stage and needs [32]. This mechanism is expected to be conserved in SARS-CoV-2 as nsp3 shares 91.8% or 86.5% sequence similarity with that of SARS-CoV according to [23, 24], respectively (Table 1). This suggests the importance of further analysis to understand the significance of the interaction on host cells.

#### PDPK1 interaction with SARS-CoV M protein

SARS-CoV membrane (M) protein is the most abundant constituent and the major player in the viral assembly giving the envelope its shape and size. It is considered the mainstay of this process due to its ability to interact with all the other main structural proteins (E, S, and N proteins) [33, 34]. The C-terminus of the M protein was found to interact with phosphoinositide-dependent kinase-1 (PDPK1/PDK1) through the pleckstrin homology (PH) domain (Table 1). This interaction was investigated via Co-IP after observing the co-localization of both M protein and PDPK1 in the cytoplasm using confocal microscopy (Table 1) [35]. PDPK1, the serine/threonine kinase, is a master kinase that phosphorylates and activates several target proteins including protein kinase B (PKB/Akt1, PKB/Akt2, PKB/Akt3). PDPK1 contributes to various pathways: cellular response towards DNA damage, insulin signaling, cell growth, proliferation, and survival, besides its crucial role in cardiac homeostasis [36]. In addition, it interacts with multiple key signaling proteins (Fig. 1C). Akt1 mediates double-strand breaks (DSBs) repair through the nonhomologous end-joining (NHEJ) pathway. It directly interacts with the catalytic subunit of DNA-dependent protein kinase (DNA-PKcs), and the complex is then recruited to the Ku-linked broken ends [37]. Akt is also critical for generating interferon (IFN)-dependent antiviral response [38]. Although the cellular consequences of the interaction are not well studied, the expression of the vesicular stomatitis virus (VSV) M protein disrupts Akt phosphorylation [39]. Similarly, the increase in the measles virus (MV) pathogenicity is owed to Akt inhibition [40]. Altogether, one could speculate that the Akt activity could be downregulated upon SARS-CoV infection. It is also very likely that a similar mechanism exists in SARS-CoV-2-infected cells, as the reported sequence similarity between SARS-CoV and SARS-CoV-2 M proteins is 98.2% or 96.4% according to [23, 24], respectively (Table 1). We particularly speculate adverse effects on DSB repair due to the compromised Akt DNA repair function.

#### DDX1 interacts with SARS-CoV viral nsp14

DEAD-Box helicase 1 (DDX1) is a member of the DEAD-box proteins, a putative RNA helicases family [41]. This helicase was initially discovered in the nucleus, where it forms the so-called DDX1 bodies [42]. DDX1 contributes to DSBs repair through maintaining the single-stranded DNA generated by end resection during homology-directed repair [43]. In addition, DDX1 was shown to have RNase activity on single-stranded RNA (ssRNA) and ADP-dependent unwinding activities for both RNA-DNA and RNA-RNA strands, suggesting a function in clearing RNA at the DSB site [42].

Through Co-IP, the host DDX1 was shown to interact with nsp14 of SARS-CoV (Fig. 1D) [44]. nps14 is a 3′–5′ exoribonuclease and a methyltransferase that plays an essential role in the high-fidelity viral replication [45]. Interestingly, the DDX1-nsp14 interaction upon IBV infection causes DDX1 translocation from the nucleus to the cytoplasm, which interferes with its important nuclear roles [44]. Since nsp14 shares 99.1% or 98.7% sequence similarity with SARS-CoV according to [23, 24], respectively, comparable consequences could also be expected for the recently identified virus (Tables 1 and 2).

**Table 2:** A summary of SARS-CoV-2 genes coding for proteins that interact with the host DDR

SARS-CoV-2 protein	Function	Host DDR proteins	Protein sequence similarity to SARS-CoV according to [24]	Protein sequence similarity to SARS-CoV according to [23]	
Spike protein (S subunit)	Host cell entry	BRCA1 (in silico)	87.0%	91.5%	
		BRCA2 (in silico)			
		p53 (in silico)			
		BRD4			
Envelope protein (E protein)	Viral replication and assembly	BRD2/4	96.1%	97.4%	
ORF8 protein	Modulating the host immune response and interferon signaling inactivation	DNMT1	45.3%	ORF8 a	70.7%
		5-LOX		ORF8 b	66.7%
ORF9b protein	Suppress antiviral innate immunity	DCTPP1	84.7%	NA	
Nucleocapsid protein (N protein)	Immune suppression	DDX1	94.3%	97.2%	
ORF10 protein	Inhibits innate immune response	CUL2^ZYG11B^	NA	ORF9b	52.4%
nsp1/leader protein	Suppress cellular protein synthesis and potent inhibitor of host gene expression and antiviral response	Polymerase α	91.1%	93.9%	
nsp5/3C-like proteinase	Processing of viral polyprotein	HDAC2	98.7%	99.7%	
nsp14	Guanine N7-methyltransferase 3′–5′ exoribonuclease (evasion of host immune response)	IMPDH2	98.7%	99.1%	

### The interaction between SARS-CoV-2 and the host DDR

Several studies have reported high-confidence physical associations between SARS-CoV-2 proteins and human cellular proteins using affinity-purification mass spectroscopy (AP-MS) [24, 46, 47]. In addition, other studies have focused on in silico prediction of viral-host interactions via bioinformatics analysis [48]. Here, we review the reported interactions between SARS-CoV-2 and DDR proteins.

### BRD2/4 interact with SARS-CoV-2 E protein

Several bromodomain proteins (BRDs) get recruited for DSB repair through chromatin-remodeling complexes. For example, BRD2 binds to the acetylated histone 4 at the DSB site to protect it from histone deacetylases. BRD2 then allows for the recruitment of a second bromodomain protein, ZMYND8, which promotes the acetylation process [49]. Additionally, BRD4 recruits condensin II to remodel the acetylated histones, thus inhibiting DDR signaling [50].

**Fig. 2:**
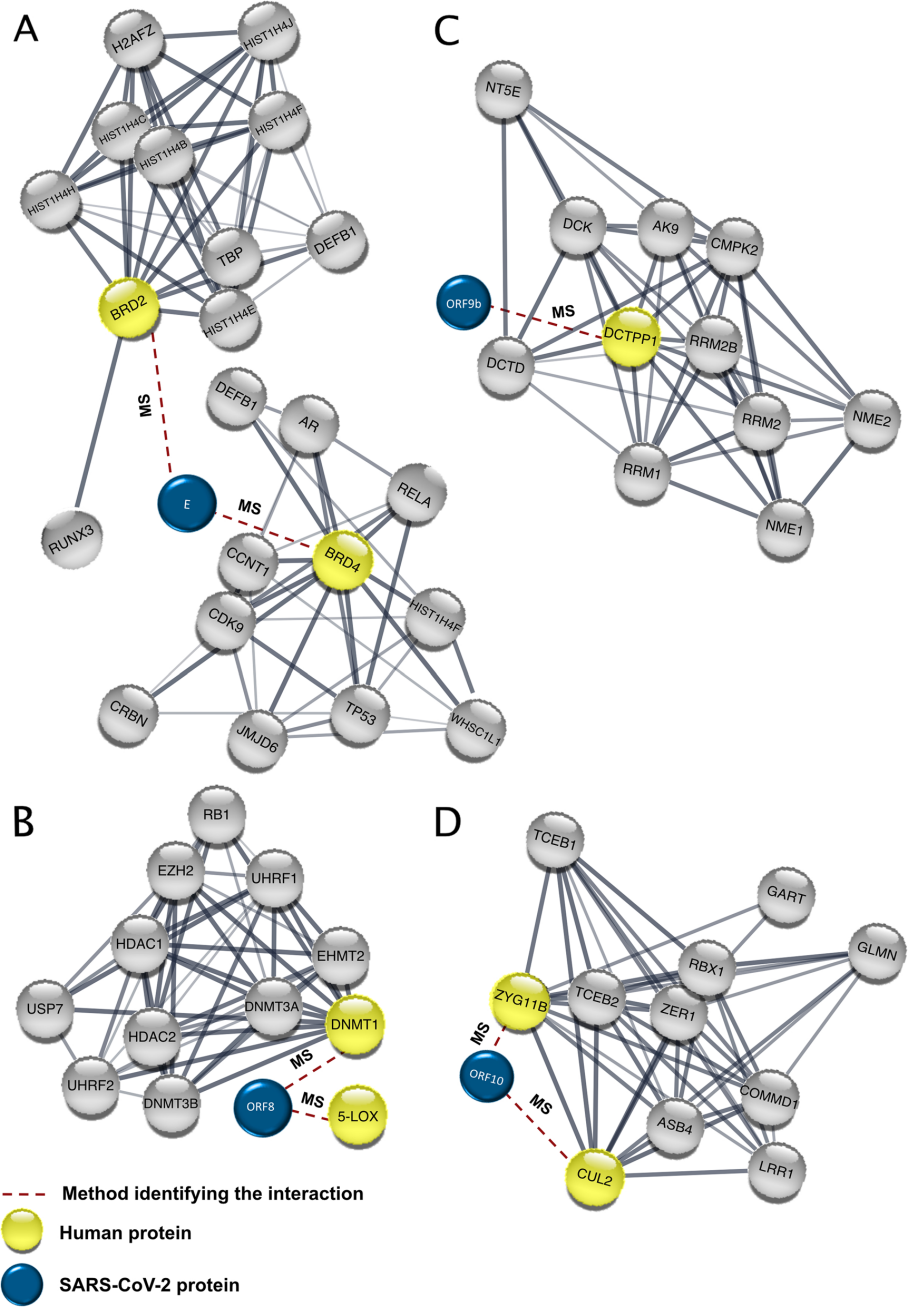
SARS-CoV-2 protein interactions with BRD2, BRD4, DNMT1, 5-LOX, DCTPP1, and CUL2^ZYG11B^. **A**-**D**) The viral proteins are linked to the interaction partner represented in a protein-protein association network retrieved from STRING [26]. MS, mass spectrometry

The SARS-CoV-2 envelope (E) protein forms an ion channel, and its C-terminus resembles the N-terminus of histone H3. Therefore, similar to H3, it was shown to directly interact with bromodomains (BRDs) (Table 2) (Fig. 2A) [24, 51]. In addition to the interaction of the viral E protein with BRDs, the spike protein of SARS-CoV-2 results in enhancement of BRD4 expression, which is a regulator for senescence mechanism. Therefore, high levels of reactive oxygen species (ROS), DNA damage, and cellular senescence were observed in the infected cell lines. Interestingly, treatment of the cells with a BRD4 inhibitor reversed the senescent phenotype [52].

Several viruses have shown direct interactions with host BRDs as well. For example, the human papillomavirus (HPV) tethers its genome to the host chromosomes through binding of the E2 protein to the host BRD4 [53]. Therefore, tackling the consequences of SARS-CoV-2 binding to bromodomain proteins can lead to promising findings and the discovery of potential viral inhibitors.

### DNMT1 interacts with SARS-CoV-2 ORF8

DNA methyltransferase 1 (DNMT1) is an essential player in the process of DNA methylation. Knocking down DNMT1 in telomerase reverse transcriptase (hTERT)-immortalized normal human fibroblasts caused an indirect defect in the MMR pathway, as a consequence of decreased levels of MutLα and MutSα complexes [54].

SARS-CoV-2 ORF8 protein interacts with DNMT1 (Table 2) (Fig. 2B) [24]. In addition, ORF8 is potentially proposed to hinder host immunity as it interferes with type-I interferon (IFN-I) signaling [55]. Moreover, ORF8 downregulates the major histocompatibility complex I (MHC-I) [56].

It was shown that hepatitis C virus (HCV) exploits both DNMT1 and DNMT3B to propagate since the HCV sub-genomic replication is inhibited via the downregulation of DNMT1 or DNMT3B [57]. Hence, we hypothesize that SARS-CoV-2 ORF8 and DNMT1 interaction might affect the human DNA repair machinery and contribute to viral pathogenicity. Further studies are also needed to determine if DNMT1 inhibitors can affect SARS-CoV-2 pathogenicity and be used as a potential therapy.

### 5-LOX interacts with SARS-CoV-2 ORF8

5-Lipooxygenase (5-LOX) is important for leukotrienes biosynthesis. Moreover, it regulates the activity of the tumor suppressor p53, which is involved in DSBs repair. p53 also regulates its ∆133p53 isoform that participates in DSB repair, via upregulating the transcription of key repair genes; *RAD51*, *LIG4*, and *RAD52* [58].

SARS-CoV-2 was shown to interact with the host 5-LOX via MS analysis (Table 2) (Fig. 2B) [24], which opens questions regarding the effect of the interaction on DDR and the viral virulence. Interestingly, high levels of 5-LOX were detected during Kaposi’s sarcoma-associated herpes virus (KSHV) infection. The interaction contributes to viral pathogenicity, and the inhibition of 5-LOX expression negatively affects the KSHV latency [59]. In the same manner, SARS-CoV-2 interaction with 5-LOX may contribute to increasing the SARS-CoV-2 pathogenicity and affect the human DDR.

### DCTPP1 interacts with SARS-CoV-2 ORF9b

ORF9b, an alternative open reading frame within the N gene of SARS-CoV-2, encodes one of the most important accessory proteins involved in impeding host immune response by acting on the mitochondria. It works on interferon deactivation via targeting the translocase of the mitochondrial outer membrane 70 (TOM70), which facilitates its evasion [60]. Interestingly, the ORF9b of SARS-CoV-2 interacts with the human dCTP pyrophosphatase 1 (DCTPP1) (Table 2) (Fig. 2C) [24]. DCTPP1 regulates the deoxynucleotide (dNTP) pool homeostasis with a higher affinity towards deoxycytidine triphosphate (dCTP) and its analogs. Furthermore, DCTPP1 preserves the nuclear and mitochondrial genomic integrity through protecting the DNA and RNA from genotoxic nucleotide analogs misincorporation [61, 62]. We propose that the host’s mitochondrial DNA (mtDNA) can suffer from damage since ORF9b localized to the mitochondria in both SARS-CoV- and SARS-CoV-2-infected cells [5].

### CUL2^ZYG11B^ complex interacts with the ORF10 of SARS-CoV-2

In SARS-CoV-2, the ORF10 protein inhibits the type-I interferons (IFN-I) signaling pathway and induces the breaking down of the mitochondrial antiviral signaling protein (MAVS). Moreover, ORF10 induces mitophagy through interacting with the mitophagy receptor Nip3-like protein X (NIX) in the mitochondria. Consequently, this inhibits the antiviral innate immune response [63]. ORF10 interacts with multiple human proteins which play a vital role in different cellular pathways [24]. Notably, it interacts with the Cullin 2 RING E3 ligase complex bearing the substrate adapter ZYG11B (CUL2^ZYG11B^) (Table 2) (Fig. 2D) [24]. CUL2 is important for the regulation of protein degradation. Therefore, silencing it significantly impairs S-phase entry and delays the recruitment of RAD51 to repair DNA via homologous recombination (HR). Even though the consequences of the ORF10-CUL2^ZYG11B^ interaction are still not clear, the ubiquitination pathways are usually hijacked by viruses for replication and pathogenesis purposes [64, 65]. For instance, the adenoviruses, human immunodeficiency virus, type 1 (HIV-1), HPV type 16, and Epstein-Barr virus (EBV) exploit ubiquitin ligases to target cellular proteins for degradation and help in viral replication [66, 67]. Particularly, adenovirus 12 was shown to utilize a CUL2/RBX1/elongin C-containing ubiquitin ligase to degrade p53 during infection and induce the ATR activator protein topoisomerase-IIβ-binding protein 1 (TOPBP1) degradation [68]. The degradation of TOPBP1 compromises DDR, as it binds SSBs, DSBs, and DNA nicks and acts as a sensor for replication stress [69, 70]. Hence, SARS-CoV-2 interaction with CUL2 might aid in viral replication and manipulation of the DDR.

### Polymerase α complex interacts with SARS-CoV-2 nsp1

**Fig. 3:**
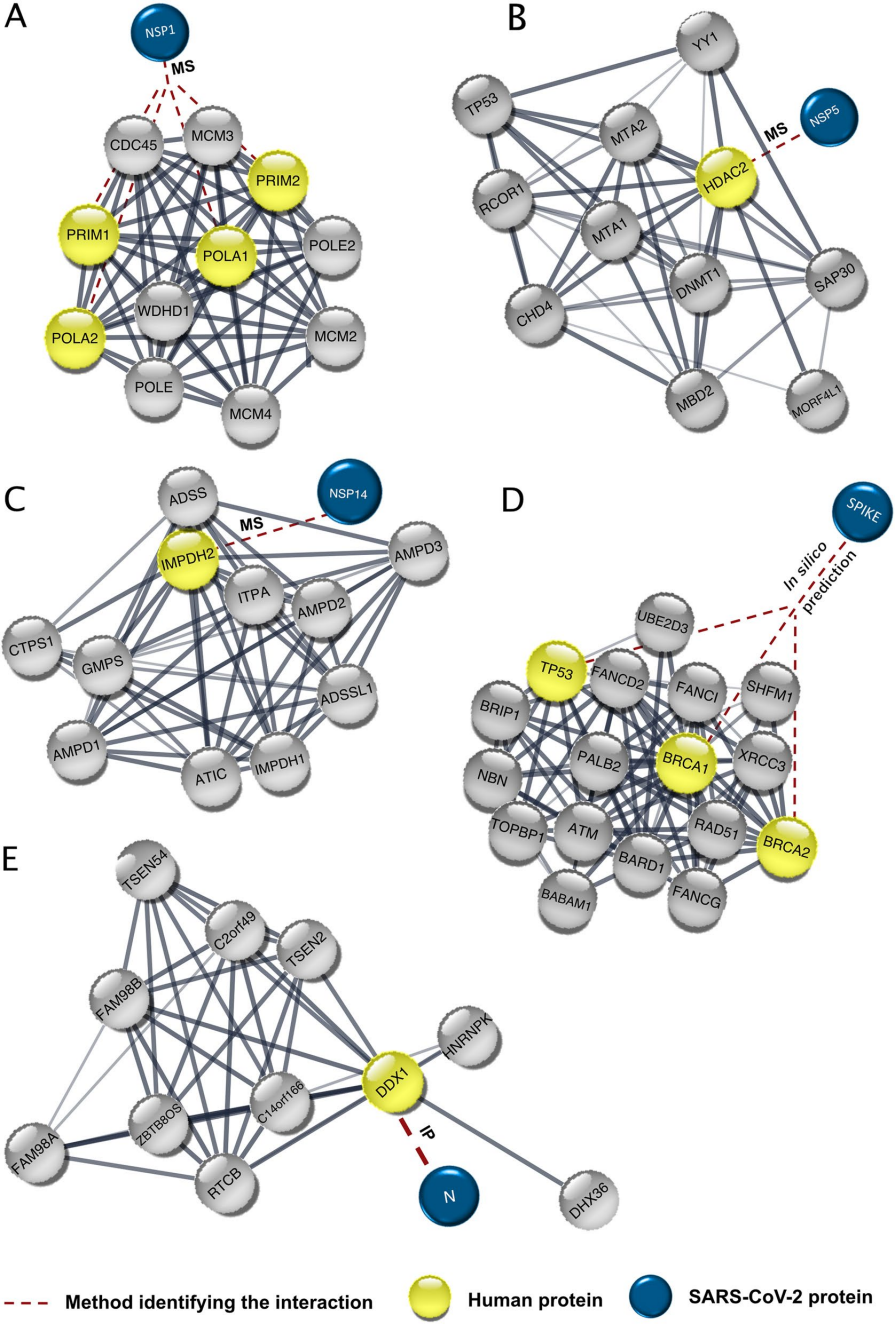
SARS-CoV-2 protein interactions with the DNA polymerase α complex, HDAC2, IMPDH2, BRCA1, BRCA2, TP53, and DDX1. **A**, **B**, **C**, **E**) The viral proteins are linked to the interaction partner represented in a protein-protein association network retrieved from STRING [26]. **D**) Viral spike protein predicted interaction with BRCA1, BRCA2, and TP53 networks. The networks of BRCA1 and BRCA2 were retrieved from STRING and merged [26]. MS, mass spectrometry; IP, immunoprecipitation

nsp1 plays an important role in regulation of viral replication and translation. It increases the SARS-CoV-2 infectivity by downregulating the host antiviral pathways, specifically the interferon pathway components. This occurs through stalling mRNA translation by blocking the ribosomal 40S subunit, reducing mRNA translation [71]. SARS-CoV-2 nsp1 was shown to interact with all four subunits of the DNA polymerase α complex: POLA1, POLA2, PRIM1, and PRIM2 (Table 2) (Fig. 3A) [24]. Since the polymerase α complex is essential for initiating DNA replication as well as NHEJ [72], we suggest that this interaction may cause replication stress along with defects in NHEJ.

### HDAC2 interacts with SARS-CoV-2 nsp5

Histone deacetylases 1/2 (HDAC1/2) localize at the DNA replication site and interact with the PCNA to ensure DNA replication efficiency [73]. During DNA damage, HDAC1/2 get recruited to promote hypo-acetylation of H3K56 at the DNA damage sites. In addition, they promote NHEJ to repair the DNA damage [74]. Additionally, HDAC regulates the ATM and p53 expression and activities affecting the DNA damage signaling [75]. A high-confidence interaction between HDAC2 and SARS-CoV-2 nsp5 was identified (Table 2) (Fig. 3B) [24]. nsp5 is a protease that cleaves pp1a and pp1ab polypeptides at eleven positions to release mature and intermediate nonstructural proteins crucial for viral assembly [76]. A cleavage site between the nuclear localization sequence of HDAC2 and its catalytic domain was predicted to be processed by nsp5; thus, the viral interaction with HDAC2 is proposed to prevent the nuclear localization of HDAC and the subsequent activation of the interferon response pathway [24]. Overall, the interaction of SARS-CoV-2 with HDAC2 could have negative consequences on the host genome integrity.

### IMPDH2 interacts with SARS-CoV-2 nsp14

Inosine-5′-monophosphate dehydrogenase (IMPDH) isoforms, IMPDH1 and IMPDH2, play an important role in cell growth regulation by catalyzing the conversion of inosine 5′-phosphate (IMP) into xanthosine 5′-phosphate (XMP) in the de novo synthesis pathway of guanine nucleotides [77]. It was found that the SARS-CoV-2 nsp14 interacts with IMPDH2, proposing a possible alteration of its function (Table 2) (Fig. 3C) [24]. nsp14 is required for SARS-CoV-2 replication fidelity and messenger RNA (mRNA) capping, contributing to the viral pathogenicity and life cycle through the control of the innate immune response and the viral genome recombination [78]. The prolonged inhibition of IMPDH causes replication stress, DNA damage, and genomic instability as a result of nucleotide pool imbalance [77]. Subsequently, we propose that nsp14 protein interaction with IMPDH can result in severe cellular defects.

### BRCA1, BRCA2, and p53 interact with the S2 subunit of the S protein

The spike (S) glycoprotein of SARS-CoV-2 undergoes proteolytic cleavage at the S1/S2 site by host furin or furin-like proteases [79]. This cleavage results in the surface subunit S1, responsible for the virus attachment to the host cell surface receptor, and the transmembrane subunit S2, which derives the fusion of the viral and host membranes and allows the release of the viral genome into host cells [80]. The interaction of the S2 subunit with p53, BRCA1, and BRCA2 was predicted in silico (Table 2) (Fig. 3D) [48]. BRCA1, BRCA2, and p53 are well-known tumor suppressor proteins. BRCA proteins participate in HR to repair DNA DSBs. BRCA1 functions upstream of BRCA2 in response to DNA damage, while BRCA2 plays a main role in the regulation of RAD51 activity in HR machinery [81]. BRCA1 was previously associated with Tat-dependent transcription enhancement of the HIV-1 infection [82]. Moreover, numerous functional activities of BRCA1 are antagonized by interacting with oncogenic HPV E6 and E7 proteins [83]. Further studies are required to confirm the interaction of the proteins in vitro and also to understand the extent of DNA damage caused by this interaction if confirmed.

### DDX1 interacts with the SARS-CoV-2 viral N protein

Similar to SARS-CoV, SARS-CoV-2 interacts with the host DDX1 but through the viral N-protein (Table 2) (Fig. 3E). As previously mentioned (“DDX1 interacts with SARS-CoV viral nsp14”), DDX1 contributes to DSBs repair [43]. The N protein-DDX1 interaction was shown to be important for viral replication [84]. Further analysis may reveal significant cellular changes as a result of this interaction.

## Conclusions

**Fig. 4:**
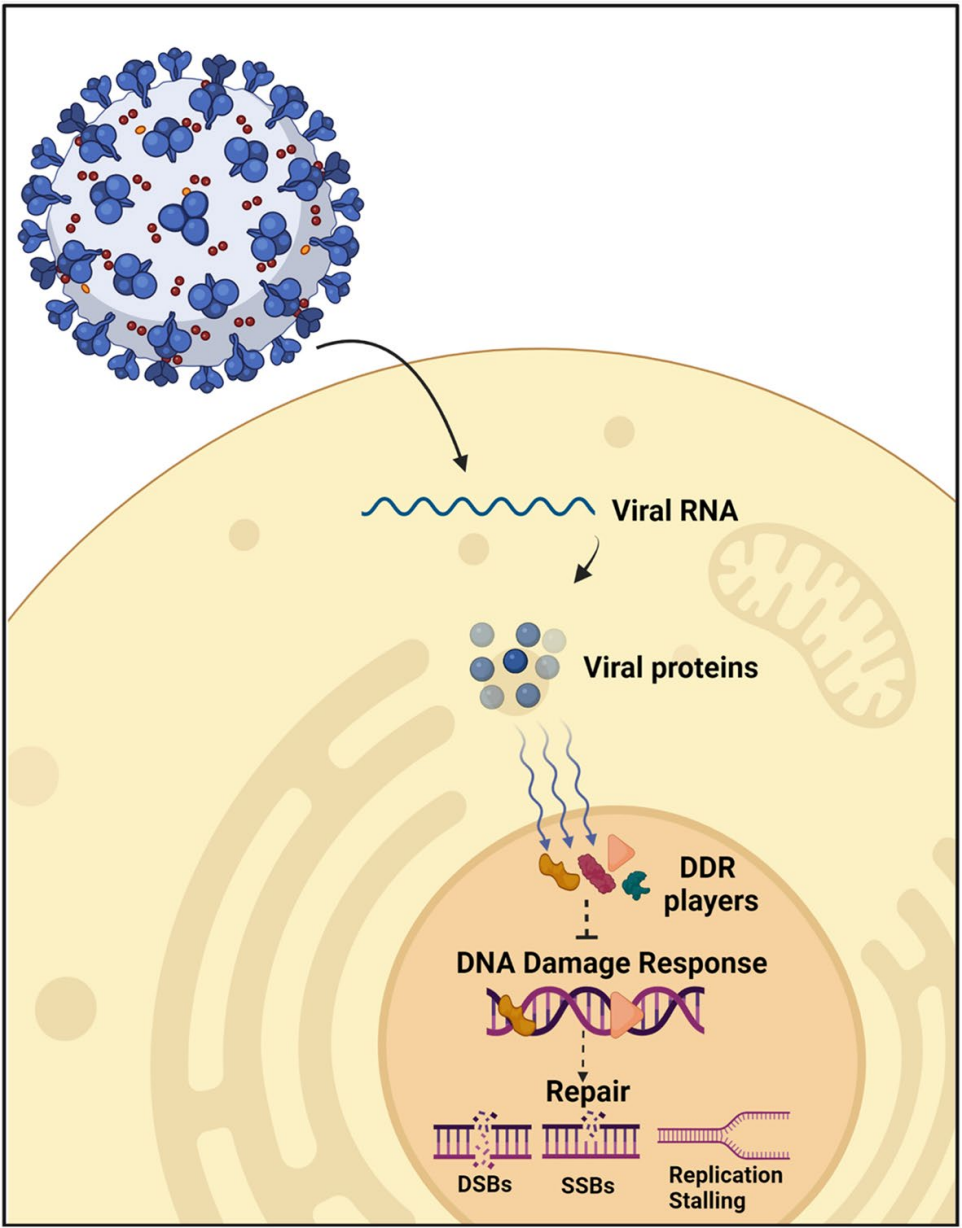
The potential consequences of viral proteins interaction with host DDR players. Upon infection of the host cells, the viral genome gets translated into proteins. Some of the viral proteins will enter the nucleus and interact with DDR associated players, potentially affecting  the cellular ability to respond to DNA damage and stimulate DNA repair. This can eventually result in accumulation of single-stranded (ss) and double-stranded (ds) breaks in addition to replication fork stalling upon encountering DNA damage. The figure is created with BioRender.com

Despite the global interest in SARS-CoV-2 research, most of the studies emphasize on the structural aspects of the viral-host interactions, with a limited focus on the molecular consequences on the host genome. Several SARS-CoV-2 proteins were reported to interact with host players associated with DDR, which can negatively impact their contribution to the repair of DNA damage (Fig. 4). The integration of SARS-CoV-2 reverse-transcribed sequences into the infected human genome was also reported to express chimeric virus-host RNAs, which affects host genome integrity [85]. Here, we reviewed the SARS-CoVs-host DDR interactions and proposed possible effects on genome stability and DNA repair (Fig. 4). We also compared the sequence similarity of homologs for both viruses and reported the DDR manipulations induced by other viruses to get more insights into the current virus behind the pandemic from previously discovered ones.

The DDR-targeting drugs are showing a great promise in combating the virus. For example, berzosertib, an inhibitor for the major DNA damage sensor ATR kinase, shows potential anti-SARS-CoV-2 activity in multiple cell types in addition to its ability to inhibit SARS-CoV replication [86]. Mapping more interactions that influence the host DDR is important for developing antiviral drugs that can target a broad range of emerging strains. As discussed, the majority of the reported interactions for SARS-CoV-2 so far were observed through high-throughput methods or in silico simulations. Therefore, future studies should invest in small-scale verification of interactions to confirm the potential use of the reported host DDR proteins as drug targets [46, 87]. Further validation of the targets using in vivo models will also be necessary to confirm the output obtained from experiments on cell lines in a multicellular context. Overall, this area of research should expand and develop to enable the discovery of novel antiviral drugs that treat SARS-CoV-2 and other viruses of similar mechanisms that can emerge in the future.

## Data Availability

N/A.
